# Supplementary material for: Multiple imputation of maritime search and rescue data at multiple missing patterns
Source: PLoS One. 2021 Jun 18;16(6):e0252129. doi: 10.1371/journal.pone.0252129 (PMC8213137; doi:10.1371/journal.pone.0252129)
Supplement: Attachment — Submitted filename: Comments.docx [file pone.0252129.s001.docx]

Well known multiple imputation method and algorithms are properly applied. Data and results clearly presented in figures.

1. In case of some variables the general sense of applying this method for SAR purposes rises questions and reservations:
1) “Death” (“Death is the number of missing or dead persons”).

What is a purpose of assessing (or receiving) number of “death” (in previous years) by statistical methods?

Who (or what kind of institution) is interested in receiving such data?

2) “Weather” (“Weather is the number of bad weather”)

To be defined what is “bad weather” – e.g. wind force higher than 8° B, 10°B, sea state, tropical storms, poor visibility?

Why do the Authors try to input missing data by statistical methods instead of checking real meteorological surface analysis existing in the shipping world and other meteorological data bases?

3) “Volume” (“Volume is the volume of waterborne transport”)

Figure 6 . Observed versus imputed Value of Volume -

What are the units? ( e.g. 75000 - individual ships observed/ analyzed?)

4) “TR” (“TR is the times of distress”)

Units again.

E.g. What is TR 200? (Figure 6)

2. Other remarks:
1) Table 1. “Safety status” to be used rather than “Security status”, as the word “Security” is used in shipping for piracy and maritime terrorism issues.
2) Figure 4 - axes x - missing labels at 30% missing rate.
3) Generally clear and understandable language, but some sentences require language corrections (including professional maritime vocabulary) as they are difficult to understand (e.g. In 2018, China had a total of 176 water traffic accidents with general and higher levels associated with transportation ships, causing 237 people missing, 83 shipwrecks, and a direct economic loss of 290 million yuan [4-5].)
