# Supplementary material for: Multiple imputation of maritime search and rescue data at multiple missing patterns
Source: PLoS One. 2021 Jun 18;16(6):e0252129. doi: 10.1371/journal.pone.0252129 (PMC8213137; doi:10.1371/journal.pone.0252129)
Supplement: Attachment — Submitted filename: Response to Reviewers.docx [file pone.0252129.s002.docx]

Response to Reviewer #1: Thank you for your valuable comments. The amendments to your comments are as followings:

**1. The authors do not reference original sources, such as seminal work by Rubin done in 1970's on multiple imputation.**

I have read many papers on multiple imputation by Rubin, such as Inference and missing data published in 1976, and Statistical analysis with missing data published in 2002, which created a precedent for data imputation. Actually, my paper is mainly about the latest applications of multiple imputation, and citation of original sources would also be helpful. Rubin's Inference and missing data published in 1960 has already been cited in the paper.

**2. This article compares the effect of DA algorithm and EMB algorithm on the processing of missing data, but it does not highlight the particularity of maritime search and rescue data.**

This paper has already explained the composition of search and rescue data when introducing the data source, and specifically analyzed the missing features of search and rescue data when constructing the missing model, and these are the main conditions required for data imputation.

**3. Please clarify your significant contributions besides utilizing existing algorithms for handling missing data.**

The main contribution of this paper is to analyze the characteristics of search and rescue data, and adopt a relatively reliable method to impute the data based on the comparison of interpolation methods, which better solves the problem of data missing in search and rescue simulation. Besides that, evaluation methods such as probability density curves and over-imputation diagnostics are used to evaluate the effect of data interpolation more accurately, which evaluate the effect of interpolation depending on the data distribution, and avoid erroneous evaluation of the effect of interpolation by averaging methods such as interpolation average. This article provides new ideas for method selection and effect evaluation of data interpolation.

**4.** **It has to be described if the algorithm used in this article is better than data imputation based on professional experience in the field of maritime search and rescue.**

Due to the difference among areas, water environments, personnel qualities and other factors, there is great uncertainty in the number of ships in distress. Consequently, it seems that the method of data interpolation using professional experience was not applied at present. This also explains the rare usage of data interpolation in the field of maritime search and rescue.

**5. The article only imputation the missing data within five years. Will it affect the results in the case of a long time series?**

Data volume is sufficient to meet the demands of multiple imputation sample construction. It will not affect the results in the case of a long time series.

**Here are some clerical mistakes:**

**1. Delete “based on” on line 45.**

"Based on" in line 45 has been deleted.

**2. Irregular chart format in table 5.**

The format in Table 5 has been adjusted.

**3. The figures are not clear.**

The picture meets the resolution requirement, and it is clear, even after a click to enlarge. If it still cannot be displayed clearly, I will upload the picture again.

Response to Reviewer #2: Thank you for your constructive amendments. The amendments to your comments are as follows:

**1. In case of some variables the general sense of applying this method for SAR purposes rises questions and reservations:**

1) **“Death” (“Death is the number of missing or dead persons”).**

**What is a purpose of assessing (or receiving) number of “death” (in previous years) by statistical methods?**

The main purpose of counting the death toll is to quantify the risk of accidents, and it also reflects the difficulty of search and rescue and the level of water traffic safety.

**Who (or what kind of institution) is interested in receiving such data?**

Government departments are more interested in these data, especially maritime management departments. These data help them understand the current difficulty of water search and rescue and whether they can complete the search and rescue mission.

2) **“Weather” (“Weather is the number of bad weather”)**

**To be defined what is “bad weather” – e.g. wind force higher than 8° B, 10°B, sea state, tropical storms, poor visibility?**

The number of bad weather in this article specifically refers to the number of days affected by typhoons, because typhoon has the greatest impact on the safety of ships at sea. Futherer, typhoon is also a comprehensive variable, containing the factors of rain, visibility, wind, etc.

**Why do the Authors try to input missing data by statistical methods instead of checking real meteorological surface analysis existing in the shipping world and other meteorological data bases?**

The data used to analyze the missing pattern in this article is the actual missing data set, and the subsequent data set used for the missing analysis is the complete data to analyze the accuracy of the imputation result. Therefore, the subsequent missing analysis of meteorological data is to evaluate the effect of imputation, and will not replace the existing data with imputed data.

3) **“Volume” (“Volume is the volume of waterborne transport”)**

**Figure 6 . Observed versus imputed Value of Volume -**

**What are the units? ( e.g. 75000 - individual ships observed/ analyzed?)**

The abscissa is the actual waterway freight volume of the month, its unit is Ton. The ordinate is the interpolation data of the observed value, its unit is also Ton.

4) **“TR” (“TR is the times of distress”)**

**Units again.**

**E.g. What is TR 200? (Figure 6)**

The abscissa is the actual times of distress in month, its unit is Time. The ordinate is the interpolation data of the observed value, its unit is also Time. TR 200 means the distress of this month is 200 times.

**Other remarks:**

1) **Table 1. “Safety status” to be used rather than** **“Security status”, as the word “Security” is used in shipping for piracy and maritime terrorism issues.**

Agree with the amendment and change "Security status" to "Safety status".

2) **Figure 4 - axes x - missing** **labels at 30% missing rate.**

Agree with the amendment and add the labels at 30% missing rate.

3) **Generally clear and understandable language, but some sentences require language corrections (including professional maritime vocabulary) as they are difficult to understand (e.g. In 2018, China had a total of 176 water traffic accidents with general and higher levels associated with transportation ships, causing 237 people missing, 83 shipwrecks, and a direct economic loss of 290 million yuan [4-5].)**

Agree with the amendment and modify the sentence in the paper.
